# Play as a Stress-Coping Method Among Children in Light of the COVID-19 Pandemic: A Review

**DOI:** 10.7759/cureus.43550

**Published:** 2023-08-15

**Authors:** Jaroslava Raudenská, Jiří Gumančík, Martin Raudenský, Alberto Pasqualucci, Marco Antonio Narvaez Tamayo, Giustino Varrassi, Alena Javůrková

**Affiliations:** 1 Nursing, Second Faculty of Medicine, Motol University Hospital, Charles University, Prague, CZE; 2 Psychology, Faculty of Health and Life Sciences, University of Northumbria in Newcastle, Newcastle upon Tyne, GBR; 3 Faculty of Education, Charles University, Prague, CZE; 4 Anesthesia and Critical Care, University of Perugia, Perugia, ITA; 5 Pain Medicine Unit, Hospital Obrero, La Paz, BOL; 6 Pain Medicine, Paolo Procacci Foundation, Rome, ITA; 7 Clinical Psychology, Third Faculty of Medicine, University Hospital Kralovske Vinohrady, Charles University, Prague, CZE

**Keywords:** children, play-based activities, play therapy, play, covid-19 pandemic, stress

## Abstract

The COVID-19 pandemic, which started in early 2020, has been a great source of stress for almost every person all around the world. However, this is particularly true for children. It is necessary to fully address the stress-related psychosocial issues connected with the pandemic, solely in children.

Play is important for children’s development, as it is a natural activity for every child. Through play and play-based interventions, children can communicate non-verbally, symbolically, and in an action-oriented manner. Therefore, play-based interventions may have the potential to be one of the coping strategies used by children who experience stress, especially during the COVID-19 pandemic.

The aim of this narrative review was to show how play-based activities could help children deal with stress related to the COVID-19 pandemic in the non-clinical population.

A systematic search of the literature in various databases was performed. The initial search provided 5,004 potentially eligible studies in various databases, and 42,201 records identified from Google Scholar. After excluding studies not meeting the inclusive criteria, nine papers were selected for this narrative review.

This narrative review showed findings that play-based activities can have a positive effect during the COVID-19 pandemic on different stress levels in the children population. Additionally, the findings of this review highlight the importance of further research and implementation of play into many aspects of children’s life.

## Introduction and background

The COVID-19 pandemic, which started in early 2020, has been a great source of stress and anxiety for almost every person all around the world [1], including healthcare professionals [2]. Since March 2020, 27% of American parents reported experiencing overall worsening of psychological difficulties, and 14% reported that their children's behavior had worsened as well [3]. The reason for such deterioration in the mental health of both parents and children might be due to rising food insecurity of 4%, or a decrease in insurance coverage of children [3]. Further, the contribution to stress might also be due to reports of regular childcare loss of 48% [3]. Furthermore, this unfortunate global event caused the shutting down of schools, workplaces, and social services, such as childcare and community programs [4,5]. Hence, children lost access to education and extracurricular activities. Moreover, socioeconomically disadvantaged children lost access to healthy nutrition provided by schools, as many disadvantaged families experienced food insecurity due to previously mentioned reasons [6]. A few families (5.6%) cannot afford meals that contain meat, fish, or a vegetarian alternative every two days [7]. The inconveniences mentioned above perpetuate restlessness due to the uncertainty about the duration and magnitude of the pandemic effect.

Many parents may struggle with their caregiving role, which causes them serious psychological distress. Consequently, parents reported toxic substance use, suicidality, self-harm, an increase in stress, and a change in their relationships with their children [8]. This generated an increased number of conflicts since the start of the social distancing measures caused by the pandemic. In tandem, the children of these parents experienced worsening mental health as well [8]. Additionally, during lockdowns, 73.8% of children frequently experienced boredom, 64.5% experienced loneliness, and 61.4% experienced frustration [9]. Irritability, anger, anxiety, and sadness were experienced by more than 30% of children.

Therefore, it is clear that there has been an enormous amount of pressure on both parents’ and children’s mental health [10]. This comes from several different indications and is caused by several different factors. It is a combination of financial, social, and consequent psychological distress. Hence, a narrative review was conducted to rigorously analyze the current literature on children’s stress and its management, and the incorporation of different forms of play, considering the events of the COVID-19 pandemic and its negative impact on all aspects of children’s life.

## Review

Methods

Aim of the Review

The aim of this narrative review was to show how play is used by children to deal with emotional baggage and stress related to the COVID-19 pandemic. In particular, we wanted to identify and analyze what has not yet been explored in relation to play activities and coping with stress during the COVID-19 pandemic. In general, we focused on an exhaustive exploration of everything actually available on the subject. The final aim was to extract the information from the research and comprehensively address its collective findings, highlighting the strengths and weaknesses, and basing recommendations for future research accordingly.

To better clarify the research aim, it is important to provide a brief definition of the main terms used, such as stress, pandemic, COVID-19, and play therapy.

Stress is a psychophysiological response to a stimulus (stressor), which induces adrenaline release. This hormonal response results in physical and psychological responses [11]. Consistent exposure to stress often has secondary consequences [12,13]. Evidence from past literature shows that experience of stress early in life is adversely associated with impairments in cognitive performance and emotion regulation [14]. Knudsen [15] argued that the experience of stress during the early sensitive period leads to altered plasticity of neuronal circuits. Furthermore, others found that anxiety and depressive symptoms have been persistently occurring in children and adolescents [16]. This may be the consequence of the risen stress levels induced by the COVID-19 pandemic, potent factors contributing to the development of both physical and mental health difficulties. Pro-inflammatory tendencies and hormonal dysregulation are described as early consequences of stress and modification of the neuronal structure of the infantile brain [17]. Additionally, Gerin et al. [18] found that stressors heighten stress levels, even for brief exposures. This results in poor social relationships, self-regulation, and an unhealthy lifestyle. Moreover, it may contribute to a poor formation of mental representation of such stressors, inducing an authentic stress response, like that caused by an actual exposure.

A pandemic is a global situation where an infectious disease spreads among a large number of individuals, usually on an international, or global level [19]. In late 2019, a COVID-19 pandemic broke out, which resulted in a global lockdown in most countries. This imposed several burdens on individuals worldwide, related to physical and psychological health, or pandemic-induced economic issues [20].

COVID-19 is an acronym for coronavirus disease 2019, which refers to the SARS-CoV-2 virus, which has broken out in late 2019, and caused a pandemic [19]. It is an airborne virus, which manifests within the first 14 days after infection. The most frequent symptoms include fever, cough, nasal congestion, alteration of smell and taste, and/or fatigue [21].

Play in children is a self-amusing and child-directed activity. It is important for children’s development, as it allows for full expression of their imagination and fantasy [22]. By having complete control over the storyline of the play, a child is free of error-related penalties. He/she is also able to fully express thoughts and feelings, and gain control over the situation [23]. In addition, play contributes to the ego development of children, forming social skills, and finding out what they like [24]. However, it also provides a view into a child’s unconscious and conscious states. The Association for Play Therapy defines play therapyas the systematic use of a theoretical model to establish an interpersonal process [25]. Trained play therapists use the therapeutic powers of play to help patients prevent or resolve psychosocial difficulties and achieve optimal growth and development. This type of therapy is used especially with children. Through play and play-based interventions, children can communicate non-verbally, symbolically, and in an action-oriented manner. That is why play therapy is one of the most important instruments of counseling and psychotherapy in preschool and young children [26].

Search Strategy and Selection Criteria

The electronic databanks searched were Scopus, PubMed, Web of Science, JSTOR, EBSCO, ProQuest, and Taylor & Francis Online. Additional records were identified through the Google Scholar search engine. The keywords were play, play therapy, children, COVID-19, stress, and mental health.

Search results were screened independently by all authors who assessed the full text of all potentially relevant publications by using a standard form with predefined eligibility criteria. Disagreements among authors were resolved by consensus. Furthermore, inclusive and exclusive criteria were used to identify relevant studies. Titles and abstracts were screened to remove duplicates. Then, titles and abstracts were assessed independently by all authors, and articles were excluded if they did not meet the predefined criteria. Lastly, the full-text versions of the remaining articles were assessed regarding inclusion and exclusion criteria. Disagreements were resolved by consensus of all authors.

Inclusion criteria were as follows: English language, the timeframe of articles within February/March 2020 to November 2022, children and stress and related mental health, and type of study design. Because we found too few relevant clinical studies, we finally included qualitative studies of any type as well. So, included studies were finally any quantitative or qualitative studies, and any publication type. Articles that were not in English or included topics regarding individuals above 17 years of age were excluded. Also, studies lacking the topic of play, play therapy, the COVID-19 pandemic, and stress were excluded.

Information of interest was extracted and collected. We used a sheet with the following headlines: author/s, title, year of publication, grant or support, number of patients and participants, age, gender, study design, assessments, and results.

Results

The initial search was conducted from September 2022 until November 2022 and provided 5,004 potentially eligible studies. Once duplicates had been removed, a total of 4,999 titles and abstracts were screened. The number of additional records identified through Google Scholar was 42,201.

After assessing the titles and abstracts, we excluded 4,959 articles. Of the remaining 40, 35 were excluded (15 studies had a different population, 10 did not assess play therapy, four did not present children less than 17 years old, and six did not deal with the COVID-19 pandemic). Finally, five articles were assessed for eligibility. One further study in this group was excluded because it did not meet the inclusion criteria; hence, only four studies of the initial group were included in this study.

Potentially eligible 42,201 records identified from Google Scholar were assessed. Studies that were not eligible were excluded: 9,450 studies had a different population, 30,001 did not assess play/play therapy, 750 did not present children, and 1,995 did not deal with the COVID-19 pandemic. Finally, five records from Google Scholar were included.

**Figure 1:**
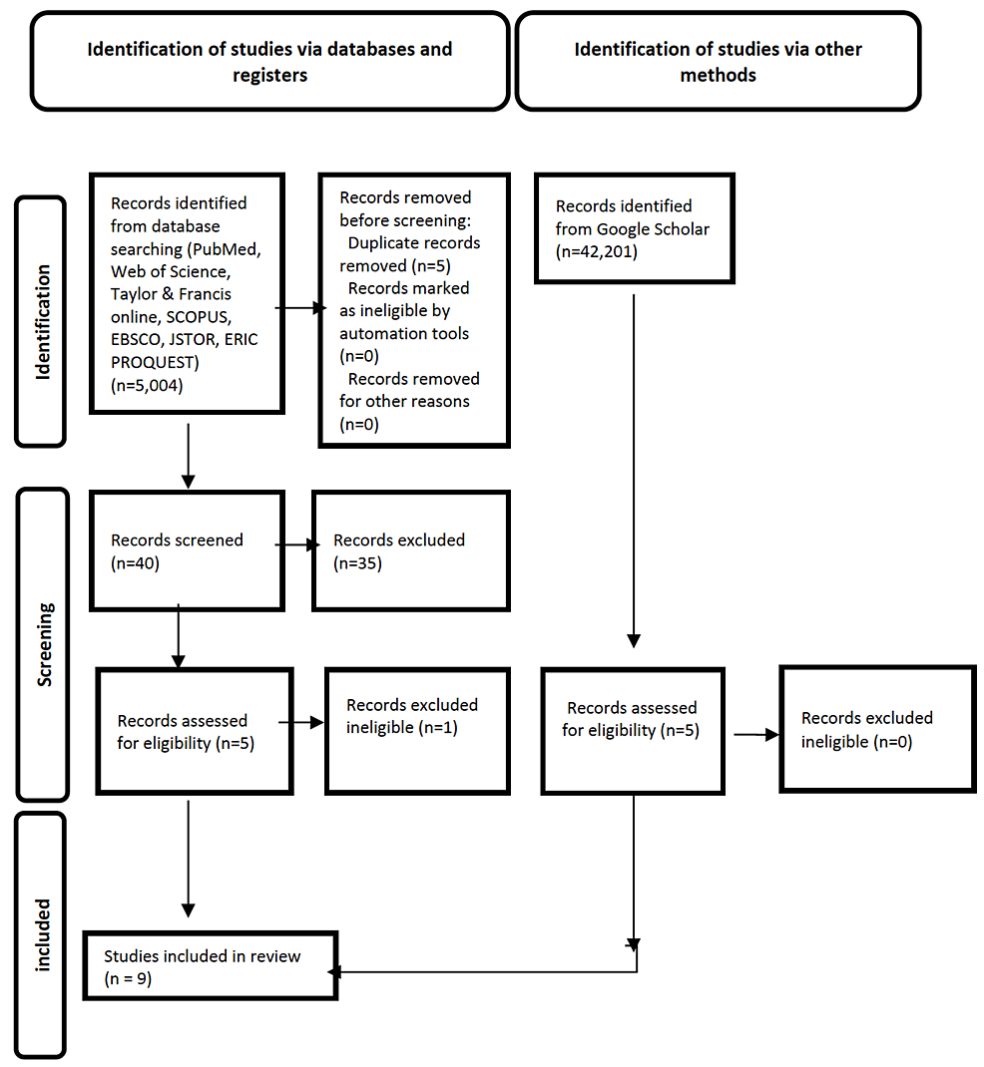
PRISMA-ScR flow diagram of study retrieval and selection PRISMA-ScR: Preferred Reporting Items for Systematic Reviews and Meta-Analyses extension for Scoping Reviews.

As a result of the inclusion criteria, nine papers in total were used for this review. The search and selection of articles were guided by the guidelines described in the Preferred Reporting Items for Systematic Reviews and Meta-Analyses extension for Scoping Reviews (PRISMA-ScR) [27]. A description of the studies is presented in Figure 1.

The quality and quantity of the publications were not large enough to meet the systematic mixed studies review (SMSR) [28-30]. That is why the result is a narrative review. The description of the studies is in Table 1.

**Table 1:** Characteristics of the nine included studies CCPT: child-centered play therapy.

Author/s, year of publication	Title	Grant or support	Aim of the article	Number of participants, age, and gender	Study design	Assessment methods	How was play incorporated into the narrative/research study	Results
Graber et al. (2021) [31]	A rapid review of the impact of quarantine and restricted environments on children’s play and the role of play in children’s health	LEGO Foundation	To investigate the impact of quarantine and isolation on children’s play and whether play might mitigate the adverse effects of quarantine and isolation on children’s health	N = 549 (527 children)	Rapid review, 15 studies	Narrative synthesis	Investigating how the quarantine and related restrictions affect play in children and young people, and how can play mitigate such effects	Playtime and behavior are impacted by social distancing measures. Play may have mitigating effects on isolating environments, which negatively impact children
Kourti et al. (2021) [32]	Play behaviors in children during the COVID-19 pandemic: a review of the literature	No	To investigate how children’s play was affected during the COVID-19 pandemic	10,313 parents with children aged 0-17 years; 2,159 children aged 0-13 years, 726 children and adolescents, 307 primary teachers	Literature review, 17 studies	Newcastle-Ottawa Scale for Cross-Sectional Studies, Newcastle-Ottawa Scale for Cohort Studies, quality assessment	Play was identified as having a possible mitigating effect on isolation impact	Play among children emerged as a coping mechanism against stress. Children involved in play and related activities showed better social skills and reduced anxiety and depressive symptoms levels
Smith et al. (2023) [33]	Virtual family play therapy: a clinician’s guide to using directed family play therapy in telemental health	No	To propose directions for adapting directed family play therapy guidelines to telemental health. It also aimed to discuss recommendations for virtual tools used to improve treatments	None	Literature review	Directive Family Play Therapy	Play was integrated into this article as a type of therapeutic approach, which can be used through virtual media as well	Directive family play therapy seems to be a valid approach even through a virtual environment. However, certain adjustments must be done, such as consultation, technology equipment testing, a more direct approach to clients, additional preparation time, and consultation prior to the session
Thompson et al. (2021) [34]	Return to play after COVID-19 infection in children	No	To educate parents of children who contracted COVID-19 on how to help them return to play and other physical activities	N/A	Commentary	N/A	Returning to play and other physical activities require medical checks of children who had contracted COVID-19 in the past. To help the process of going back, recommendations were provided on what steps to take	Depending on the level of impact COVID-19 had on the children, parents should consider the pace at which their children are going to return to play. Some require a slower and more gradual transition process, whereas some don’t. Therefore, parents should reach out to their general practitioner and consider vaccination if their child is above 12 years of age
de Lannoy et al. (2020) [35]	Regional differences in access to the outdoors and outdoor play of Canadian children and youth during the COVID-19 outbreak	ParticipACTION Research Advisory Group (RAG)	To examine the impact of different regional policies regarding COVID-19 on outdoor play among children in Canada	N = 1,472 parents of children aged 5-17 years	Commentary	Questions about children’s physical activity, sleep, sedentary time, time spent outdoors and in outdoor play	Play, especially outdoor play was considered to be an important factor, which promotes better physical and psychological health. It is also important for social interactions and a sense of control and own agency	Regions with the highest COVID cases had the most restrictions on outdoor access. Hence, outdoor play also decreased, which caused some damage associated with psychological and physical well-being
Moss et al. (2020) [36]	An introduction to child-centered play therapy	No	To introduce the basic principles and steps during the process of child-centered play therapy, partially in the context of the current COVID-19 pandemic	None	Literature review	Principles of CCPT	Description of CCPT, its principles, steps, and process. Practicing CCPT during the COVID-19 pandemic was considered	Play is considered to be the most valued form of children’s expression. It also uses the child’s level of understanding. Therefore, despite the economic and environmental hardships, CCPT should be used. Regarding COVID-19, there have been challenges encountered, some of which may have made it difficult to practice CCPT, both from the therapists’ and clients’ perspectives. Logistical precautions must be taken to maintain the therapeutical standards
Moore et al. (2020) [37]	Impact of the COVID-19 virus outbreak on movement and play behaviours of Canadian children and youth: a national survey	ParticipACTION Research Advisory Group (RAG)	Examination of the immediate impact of COVID-19 restrictions on children’s play behavior and movement	Parents (N = 1,472) of children aged 5-11 years or youth (12-17 years)	Survey study	A survey assessing immediate changes in child movement and play during COVID-19, family demographic	Play was viewed as an activity, which is important for children, and how COVID-19 limited access to this activity	COVID-19-related restrictions have impacted children’s and youth’s access to play and movement activities. Despite alternatives being provided by parents, these restrictions impacted movement behaviors in a negative way
O’Keeffe et al. (2021) [38]	‘Uncharted territory’: teachers’ perspectives on play in early childhood classrooms in Ireland during the pandemic	Postgraduate scholarship by DCU Education Trust	To examine the attitudes of early childhood teachers on the role of play in early childhood education, during the COVID-19 pandemic	N = 310 teachers (N = 10 men) of children aged 4-7 years	Survey study	A 42-item survey, which asked for opinions about play integration during pandemic	Play was integrated as a pedagogical strategy that supports children’s resilience	It was found that teachers agreed on play being one of the most important tools in regard to supporting children’s socio-psychological development, learning, and transition back to schools
Thibodeau-Nielsen et al. (2021) [39]	Child adjustment during COVID-19: the role of economic hardship, caregiver stress, and pandemic play	Partial support by the College of Human Environmental Sciences and the Department of Human Development and Family Science at the University of Missouri	To understand how economic hardships caused by the COVID-19 pandemic impacted children’s emotional well-being and development, and how this varied based on engagement in pandemic-related pretend play	N = 99 primary caregivers of children aged 3-6 years	Online survey study	Questionnaire regarding COVID-19 economic hardships, Perceived Stress Scale [40], Child Behavior Checklist Parent-Report Form [41], Early Years Toolbox [42], pandemic play frequency indication form	Play was considered as a possible solution for protecting children from the adversity effects of the COVID-19 pandemic-related issues	Pretend play has protective effects on children’s and caregivers’ well-being. This is by addressing the stress of caregivers during COVID-19 pandemic-related hardships, as well as supporting children in using pretend play as an outlet

Discussion

The analyzed articles employed qualitative methodology; in particular, literature review [31-36] and quantitative design (Table 2) [37-39]. Articles employing a quantitative design [37-39] involved participant ages ranging from 0 to 17 years, and sample sizes ranging from 10 to 10,313. Studies were conducted in the USA [33,34,36], Canada [35,37], Ireland [38], Italy [39], Greece [32], and the United Kingdom [31]. One study conducted research with children [31], four studies conducted research with families [32,35,37,39], and one study was conducted on teachers [38]. Three studies did not provide information regarding the sample size [33,34,36]. Studies were conducted either as online surveys [35,37-39] or at universities [31-34,36].

**Table 2:** Studies with quantitative and qualitative design

Studies with qualitative design (N = 6)	Studies with quantitative design (N = 3)
Graber et al. [31], Kourti et al. [32], Smith et al. [33], Thompson et al. [34], de Lannoy et al. [35], Moss et al. [36]	Moore et al. [37], O’Keeffe et al. [38], Thibodeau-Nielsen et al. [39]

The articles that employed a quantitative design [37-39] involved several ways through which children cope with stress. For example, to employ healthy movement behaviors, such as bike riding, walking, or scooting, which should be facilitated by parents. Parents should also co-participate in these play-based activities. These should last at least 60 minutes each day. Leisure screen time should be limited to at least two hours a day, substituting screen time with play wherever possible.

Next, it has been suggested that it is important to address caregivers’ stress first, and in parallel, children should engage in pretend play (i.e., imaginative, make-believe, or fantasy play). The findings suggest that pretend play provides children with a safe environment where they have control over the situation and can fully express their feelings, thoughts, and emotions about the hardships of the pandemic. Overall, pretend play is thought to have a cathartic effect on children, helping them cope with stress induced by the pandemic. Lastly, surveyed teachers felt strongly about employing play in early childhood education, as they believe it helps with the socio-emotional development of children, as well as managing stress. This was also recommended to parents who should support and engage children in play during lockdowns to support their mental health as well as stress-coping abilities.

All the studies that employed a quantitative design involved play in a non-therapeutical environment. One of them involved the use of pretend play [39]. The remaining two studies recommended using play; however, did not define what type of play in particular [37,38].

The aforementioned articles described quarantine as a means of social distancing, which helps to slow down the spread of the virus. This was in the form of physical distancing for at least two meters, staying at home, a limited number of people per gathering session, and a temporary closure of public spaces, including schools and workplaces. Play was recommended as a way for children to escape and cope with the stress induced by the pandemic and consequent distancing measures.

The publications using a qualitative methodology were primarily literature reviews [32,33,36], commentaries [34,35], and one rapid literature review [31]. Kourti et al. [32] focused on the differences between indoor and outdoor play and how the frequency of these was influenced by the COVID-19 pandemic. Their results show that outdoor play not only contributes to improvements in children’s immunity, but also facilitates their connection with nature, curiosity, and improves sleep regulation.

Furthermore, different types of play that were preferred by children were described. These were active games, board games, videogames, fantasy games, educational games, skills games, and games that focused on helping others and promoting solidarity.

Moss et al. [36] focused on child-centered therapy. Additionally, play materials, methods, and environmental settings were considered. Play materials (toys) used during therapy should adapt to the child’s developmental stage. They should be safe and hard to break, as well as help elicit emotional and creative responses. In regard to the environment, low noise and pollution is important for the child and the therapist to not get distracted. It also should consist of comfortable furniture and appealing surroundings, such warm design of the room, good acoustics, and water access. Moreover, a design that would provide confidentiality and privacy is important, as children should feel confident with the therapist.

Smith et al. [33] discussed the use of directed family play therapy (DFPT) in regard to tele-mental health (TMH). Despite the social distancing measures, restricting the conduct of DFPT in person, using the virtual method appears to be just as effective, as long as the therapist takes steps toward adjusting the process for the online environment. For patients, the main advantages of using electronic forms of communication are accessibility, time convenience, and reachability to a wider range of specialists that might suit some families better than others. Furthermore, families reported feeling less stigmatized, safer, and more comfortable in their own homes. Additionally, they felt less stressed due to traveling to the place of therapy. Younger generations are more open and comfortable with using virtual means of communication with their therapists.

De Lannoy et al. [35] discussed the impact of restricted access to outdoor play on children’s and youth’s mental and physical health. Active outdoor play was considered to be important for the same reasons stated by others [32]. To help children to cope with stress related to the COVID-19 pandemic, governmental policies should consider leaving green spaces and playgrounds open, as promoting outdoor play greatly contributes to improving children’s mental health.

Graber et al. [31] discussed how quarantine and restricted environments impacted the mental health of children as well as their play routines. It was clear that children not provided with appropriate environments for play were negatively affected. Due to children being bound to hospitals, refugee homes, or other unwelcoming places, they had a hard time adjusting to the changes in social distancing measures. Due to this, children lacked opportunities for sociability, which is important for stress resilience and protection of the children’s mental health due to isolation, with potentially serious consequences for their psychological development.

Lastly, Thompson et al. [34] provided commentary on how to help children to return to play after the COVID-19 pandemic. The article discussed the struggles of returning to normal due to the possible negative long-term effects of the disease on their health. Reportedly, some children would have a fairly fast process of return, while others might require a slower, more gradual process. This is especially challenging for athletic children, who actively engage in sports. Hence, parents should consistently monitor their children and report to their general practitioner whenever necessary. In summary, two studies discussed play from a therapeutic point of view [33,36]. Four studies discussed play from a therapeutic perspective [31,32,34,35].

All articles that employed a qualitative methodology describe quarantine as a means of limiting physical contact with the aim of reducing the spread of the disease. The role of play in quarantine was described as a way for children to communicate their feelings, emotions, and worries regarding the unprecedented circumstances, as well as a way to gain control over the situation. In addition, play was also described as beneficial to children’s mental and physical health. Table 3 shows the description of play, play activities, and play approach in coping with stress during COVID-19.

**Table 3:** Description of play, play activities, and play approach in coping with stress during COVID-19 in included studies

Author/s, year of publication	Qualitative/quantitative design	Study design	Play-based activities	Play approach psychotherapy	Play-based description activities in quarantine	Toys/toy material
Graber et al. (2021) [31]	Qualitative	Rapid review, 15 studies	Creative, expressive activities that are socially connecting children	Art-based and creative activities	Using tablets for communication and online play with friends to facilitate communication	Drawings, tablet technology
Kourti et al. (2021) [32]	Qualitative	Literature review, 17 studies	Outdoor/indoor play, computer games, playing with toys, online interaction (yoga, gym sessions)	N/A	Games, board games, play with manipulatable toys, fantasy play, pandemic-related play	N/A
Smith et al. (2023) [33]	Qualitative	Literature review	N/A	Non-directed and directed-family play therapy	Virtual family play therapy	Real-life figures, animals, baby dolls, games, art materials, small vehicles, and other objects around such as stones and sticks
Thompson et al. (2021) [34]	Qualitative	Commentary	Sports and athletic activities	N/A	N/A	N/A
de Lannoy et al. (2020) [35]	Qualitative	Commentary	Outdoor and indoor play activities	N/A	Indoor play increased as outdoor decreased	N/A
Moss et al. (2020) [36]	Qualitative	Literature review	Child-centered play	Child-centered play therapy	Live-time video-based play therapy sessions, virtual methods of play, mini play-therapy kits for families	Doll family, doll house, baby bottle, puppets, cars, money, register, food, kitchen, phone, etc.
Moore et al. (2020) [37]	Quantitative	Survey study	Leisure hobbies, online health and physical activity apps, outdoor play	N/A	Creative indoor activities, swap screen time wherever possible	N/A
O’Keeffe et al. (2021) [38]	Quantitative	Survey study	Dramatic play, outdoor play, and play in general	N/A	Play with parents, indoors and outdoors where possible	N/A
Thibodeau-Nielsen et al. (2021) [39]	Quantitative	Survey study	Pandemic-related play	Play therapy, pretend play	Re-enacting pandemic-themed situations through, pretend play with parents (imaginative, make-believe, or fantasy play)	N/A

Graber et al. [31] report that stress is induced in children by isolation and environments that are stress-inducing, such as refugee camps, immigration detention, or hospitals. Due to social distancing measures, these factors are amplified, as children cannot engage in play outside and have to adjust to the temporary limitations. This, however, poses challenges. Therefore, recommendations for stress management and adaptation to new circumstances through ways in which children play are provided.

Kourti et al. [32] found that despite children being able to find a way to socialize amid quarantine via video calls, they still reported missing their friends. This is a stressful situation, as social gatherings of any kind were limited throughout the pandemic. Therefore, to help alleviate stress, parents, teachers, and health providers should cooperate to create and maintain a healthy play routine both indoors and outdoors. Hence, others suggested that the top priority of health policies and governmental bodies should be keeping green and play spaces open as much as possible, as outdoor play is important for managing mental and physical health [35].

Additionally, it was urged to promote play and outdoor physical activity as it is considered to be one of the possible strategies to battle stress [37]. Furthermore, parents should keep being creative and initiative when providing play opportunities. They should also engage in these plays, as it has been found to reduce stress.

Lastly, leisure activities and screen time should be limited to a maximum of one hour per day. From the therapeutical standpoint, Moss et al. [36] suggested ways of cognitive-behavioral play therapy (CBPT) application by therapists. This involved not only managing anxiety and behavioral issues but also stress management, which is an inseparable part of CBPT. Overall, it provided therapists with information on how to select the appropriate environment and materials (e.g., toys), and described the process and steps involved in a child-centered therapy. When actively engaged, successful stress management would be one of the outcomes of the therapy sessions. One of the studies described play as a natural way for children to handle and deal with stress and stressful situations [38].

Upon pedagogical recommendations obtained by the interviewed pedagogues, children shall be supported in play due to its potential to manage stress and give a feeling of control over stressful situations. In addition, play can support children’s imagination and emotional expression. This is particularly important for stress management. Since the COVID-19 pandemic uncovered several issues with therapy delivery, Smith et al. [33] discussed managing stress through the use of TMH, which should be applied to therapies during the pandemic period. Since people cannot physically undergo therapy due to the spreading of the virus, TMH is an effective alternative for therapeutic approaches when adjusted to the patients in terms of technical, logistical, and communicative aspects. TMH allows for conducting therapy via an online environment, which is accessible to many nowadays. This helps both the therapist and the patients avoid the stress associated with transport, traffic, and time management. Furthermore, it helps manage stress induced by social exposure, which is experienced by individuals who suffer from a phobia of public spaces or leaving their houses, perhaps due to the pandemic.

Thematically, Thibodeau-Nielsen et al. [39] discussed topics for CBPT in regard to the COVID-19 pandemic. This study suggested that stress associated with COVID-19 is best dealt with through pandemic-related play themes. Another means, which is stress alleviating, is spontaneous pretend play. This is because play gives children a sense of control over the situation, which provides a safe space for expression of feelings and emotions. By re-enacting certain situations, there is a supposed purifying effect on the psyche of the children. Another important factor to consider is the caregivers’ stress [2], which when managed can also provide a healthier environment for the child, free of exogenous stressors.

Lastly, it is important to consider suggestions for the post-pandemic period when individuals return to normal work-life patterns. Hence, Thompson et al. [34] recommended the parents to be cautious when returning their children to school and athletic play activities. As this may impose a series of factors inducing stress, the best management would be to follow medical and governmental guidelines regarding regular testing and medical examination to avoid further delay of return.

Graber et al. [31] reported that the COVID-19 pandemic was found to be an isolating and stressful event, which limited the amount of time spent and dedicated to outdoor play. This was mostly due to the need for quarantine and social distancing measures. Furthermore, changes in children’s play behavior had been noticed. However, a need for research was indicated, as play was an important factor in mitigating some of the negative effects of isolation.

Similarly, others stated that play is important for children and their mental health [35]. Apparently, this is because it provides them with a sense of control in these uncertain times. Due to the self-isolation, quarantine, and social distancing measures, most of the outdoor spaces dedicated to children to play were closed down to stop the spread of the virus. Since this limited the children in outdoor play, it had a negative impact in regard to their mental and physical health. Hence, it was recommended to the governmental authorities that keeping these spaces open should be the utmost priority. As a consequence of this, the findings of Moore et al. [37] showed that the COVID-19 outbreak had a significant impact on children’s play behaviors, physical activity, and sedentary behaviors. Overall, physical activity decreased with potentially serious consequences on general health and an increase in metabolic diseases. Consequently, this contributed to sleep rates in children.

Quarantine limited overall activity and perpetuated unhealthy behaviors. The positive effects of this were parental engagement and the adoption of new hobbies. Additionally, Kourti et al. [32] found that children showed less outdoor play and physical activity; however, they compensated for it via indoor play and video games. Moreover, play, especially pretend play, was seen to contain themes of COVID-19. This was subsequently seen as a crucial contribution to children’s mental health and well-being, despite the negative effects quarantine had on their habits and play behaviors.

It was reported that the pandemic and related distancing measures brought economic hardships and other concerns to caregivers [39]. In tandem, this negatively influenced children and their mental health, well-being, and self-regulation. Play was seen as a means of alleviating the children from stress by engaging in pretend play with themes of the pandemic. Apparently, this worked by weakening the association between the caregivers’ stress and the children’s emotional distress. In regard to the worries due to the COVID-19 infection, Thompson et al. [34] reported that this might interfere with children’s return to play. As some children might require an individual approach when being brought back to normal life, it is important to follow medical advice. Play is important, especially for athletic children; therefore, parents can help children by keeping in touch with their general practitioner and reporting their child’s state.

As previously stated, the quarantine measures limited everyone in regard to the logistics of their lives. This also pertains to therapies. Hence, Smith et al. [33] recommended the use of TMH when conducting directed family therapy. This would overcome the logistical issues as well as other stressful factors induced by the pandemic. Via TMH, all therapies, play therapies included, are just as effective, when following certain adjustments. Moss et al. [36] discussed the use of child-centered therapy. Since the quarantine and pandemic have imposed several challenges regarding the conduction of child-centered therapy, therapists must bear in mind to be flexible when providing therapy in times of social distancing. This means keeping materials such as toys disinfected, conducting sessions in alternative formats such as video calls and videoconferencing, or implementing play therapy elements into the online environment. Lastly, O’Keeffe et al. [38] interviewed teachers who were found to stress the importance of play during quarantine, and especially, during the transition back to school. They strongly believed that play helps with socio-emotional development and facilitates children’s learning, despite the concerns regarding the implementation of play in regard to COVID-19.

All included studies in this narrative review agreed on play and play therapy having a strong positive impact on children’s mental health and well-being. Using toys, children can have a sense of self-control, be able to facilitate their emotions, and focus on current issues. This is helpful when dealing with sensitive topics such as isolation, uncertainty, and/or depression. The pandemic-themed play had a positive impact on children’s distress caused by their caregiver’s own stress [39]. Furthermore, this provided protection from COVID-19-related stressors.

Spontaneous pretend play was also found to be particularly useful in coping with stressful experiences. The advantage is in providing a child with a sense of control over the situation and the freedom and spontaneity of emotional expression.

Outdoor play has an important role in connecting children with nature [32]. In addition, their physical health improves as well, as play in nature boosts the immune system, providing an advantage in combating the COVID-19 spread. Similarly, play is important for children’s mental health and stress management [32,36,39]. Time spent playing outdoors strongly supports and contributes to less sedentary time, better sleep, immunity boost, and improved mental health [35,37]. Hence, play is advantageous in promoting better physical and mental health during COVID-19. Additionally, it provides a grounding in times of uncertainty by giving children a sense of control over the unprecedented situation.

Graber et al. [31] expanded upon this knowledge by focusing on the mitigating effects of play on the negative impacts of global restrictions. It provides socialization, which is a crucial point of focus in times of isolation. Furthermore, it also allows for a flow of creativity, connectivity, and the development of social skills, which are important for children, given their developmental stages. Lastly, it helps with emotional expression, feelings of depression, and anxiety, and gives the children the opportunity to reflect on lived experiences.

Teachers’ viewpoint on the importance and advantages of play during COVID-19 has resulted very informative [38]. Teachers put importance on the use of play in class and at home. This is because play is seen as a learning facilitator. They also agreed on play being important for children’s social and emotional development, particularly in times of uncertainty imposed by COVID-19.

Lastly, the advantages of using play via TMH also resulted useful [33]. Reportedly, play allows children to express their imagination through the symbolic use of toys and the environment. They can use their own "language" and form expressions, without having to "translate" this to the adults’ ways of communication. The benefits of performing this online are that a therapist is able to see the family in their natural environment, and therefore, be able to observe their natural behavior. Other benefits are in the reachability of the therapists by families with precarious access to places (e.g., living in rural areas).

Limitations

This narrative review has some limitations. Firstly, different types of studies with quantitative and qualitative designs were necessarily put together. Quantitative studies reported different types of children's populations and/or their parents. Also, for the paucity of data, we put together different types of play interventions: play, play-based intervention, and play therapy approaches. For such an inhomogeneous group with different interventions and different research designs from qualitative to quantitative, it is difficult to compare the potential of play-based intervention for coping with stress during the COVID-19 pandemic. The stress was in general analyzed at a non-specific level in the studies described and included. Moreover, the research was performed only on English-written publications, not in other languages. Deeper research also focused on other languages could provide interesting results. It should further be mentioned that the population was not clinical. Further, in the included studies, stress levels, the effect of activities, or other levels of mental health (anxiety, depression) were not reported.

The study also has certain strengths. It has evidenced that there is scarce relevant literature, which would describe and analyze the relationship between play-based interventions and stress management during the COVID-19 pandemic. There is still a substantial lack of common consensus on which type of play, play-based activity, or play therapy can provide the best results when coping with the stress related to the COVID-19 pandemic, and the associated emotional and behavioral problems in children.

Future research and clinical recommendations

As a further topic for research, it would be interesting to focus on the quantitative research of therapeutic approaches to play in a specific pediatric population during the COVID-19 pandemic or other stressful situations. Future research could focus on the therapeutic use of the play led only by licensed psychotherapists. That is why it would also be interesting, in further research, to focus on how the qualifications of a licensed psychotherapist and a play therapist affect the participants' experiences with play-based interventions and play therapy.

It must be also confirmed that the flexibility of the application of different play-based approaches/activities/therapies and the erudition of the play therapist and psychotherapist are important. Furthermore, it can be demonstrated in future research that the integrated use of multiple play approaches can also have a particular advantage in coping with stress.

It is also good to offer flexibility in different play approaches due to the developmental aspect of different age populations. Otherwise, play activities in coping with stress will be given to preschool children, differently to school children, and differently to adolescents. Therefore, play, play therapy, and play-based activities must be always adapted to the developmental aspect of children's age. The specific development of social, cognitive, behavioral, and emotional skills is always associated with the developmental aspect of children.

## Conclusions

This narrative review analyzes and synthesizes various findings and represents one of the first steps to provide a clear overview of which play-based activities are more useful. This is in regard to successfully coping with stress among children during the COVID-19 pandemic or other stressful situations associated with social contact restrictions and home isolation.

Play, play-based activities, play behavior, and play therapy have a strong positive impact on children’s mental health and well-being. The aim of this narrative review was to show how the use of play and play-based activities among children can help deal with emotional baggage and stress related to the COVID-19 pandemic. The negative experiences of children who experienced play-based activities during the COVID-19 pandemic are associated with passive indoor play activities, techniques, and behavior. The current study highlights that engaging in indoor play may have positive effects on the virus spread reduction; however, it also induces somatic-related issues, such as heightened blood pressure, decreased immunity, or obesity.
